# Potential impact of introducing the pneumococcal conjugate vaccine into national immunisation programmes: an economic-epidemiological analysis using data from India

**DOI:** 10.1136/bmjgh-2017-000636

**Published:** 2018-05-09

**Authors:** Itamar Megiddo, Eili Klein, Ramanan Laxminarayan

**Affiliations:** 1 Department of Management Science, University of Strathclyde, Glasgow, UK; 2 Center for Disease Dynamics Economics and Policy, Washington, District of Columbia, USA; 3 Department of Emergency Medicine, Johns Hopkins University, Baltimore, Maryland, USA; 4 Princeton Environmental Institute, Princeton University, Princeton, New Jersey, USA

**Keywords:** pneumonia, pneumococcal disease, vaccines, mathematical modelling, health economics

## Abstract

Pneumococcal pneumonia causes an estimated 105 000 child deaths in India annually. The planned introduction of the serotype-based pneumococcal conjugate vaccine (PCV) is expected to avert child deaths, but the high cost of PCV relative to current vaccines provided under the Universal Immunization Programme has been a concern. Cost-effectiveness studies from high-income countries are not readily comparable because of differences in the distribution of prevalent serotypes, population and health systems. We extended IndiaSim, our agent-based simulation model representative of the Indian population and health system, to model the dynamics of *Streptococcus pneumoniae*. This enabled us to evaluate serotype and overall disease dynamics in the context of the local population and health system, an aspect that is missing in prospective evaluations of the vaccine. We estimate that PCV13 introduction would cost approximately US$240 million and avert US$48.7 million in out-of-pocket expenditures and 34 800 (95% CI 29 600 to 40 800) deaths annually assuming coverage levels and distribution similar to DPT (diphtheria, pertussis and tetanus) vaccination (~77%). Introducing the vaccine protects the population, especially the poorest wealth quintile, from potentially catastrophic expenditure. The net-present value of predicted money-metric value of insurance for 20 years of vaccination is US$160 000 (95% CI US$151 000 to US$168 000) per 100 000 under-fives, and almost half of this protection is for the bottom wealth quintile (US$78 000; 95% CI 70 800 to 84 400). Extending vaccination to 90% coverage averts additional lives and provides additional financial risk protection. Our estimates are sensitive to immunity parameters in our model; however, our assumptions are conservative, and if willingness to pay per years of life lost averted is US$228 or greater, then introducing the vaccine is more cost-effective than our baseline (no vaccination) in more than 95% of simulations.

**Key questions**

**What is already known?**

- *Streptococcus pneumoniae* was responsible for an estimated 105 000 pneumonia deaths in India in 2010, in addition to causing meningitis and other forms of invasive disease.
- The pneumococcal conjugate vaccine (PCV) greatly reduced disease burden in high-income countries (HICs); however, the effectiveness and impact of PCV (including serotype replacement) varied significantly between countries, and it is significantly more expensive than other vaccines in India’s Universal Immunization Programme (UIP).
- To circumvent the paucity of information on the vaccine’s effectiveness in low-income and middle-income countries (LMICs), economic analyses of PCV in LMICs typically assume similar effectiveness as in HICs.

**What are the new findings?**

- The local distribution of dominant serotypes, host population characteristics and behaviour, and vaccination programmes affect the vaccine’s effectiveness.
- Despite uncertainty, we project that the vaccine will avert a significant number of deaths, provide financial risk protection for poor populations and deliver value for the cost as assessed by WHO’s cost-effectiveness guidelines.

**What do the new findings imply?**

- Economic analysis should consider local context and the dynamics of *S. pneumoniae* transmission and serotype replacement within that setting.
- Given our conservative assumptions and our projections of PCV13 effectiveness and impact in India, we recommend including PCV13 in the UIP, though we caution that existing data gaps remain and the vaccine’s effectiveness should be continuously monitored as it is rolled out.

## Introduction

*Streptococcus pneumoniae* was responsible for an estimated 393 000 (95% uncertainty interval 228 000 to 532 000) child pneumonia deaths globally in 2015, with nearly all mortality occurring in low-income and middle-income countries (LMICs).^1^ The introduction of a seven-valent pneumococcal conjugate vaccine (PCV7) in the early 2000s greatly reduced disease incidence and hospitalisation in high-income countries (HICs), by reducing invasive pneumococcal disease (IPD), which occurs when *S. pneumoniae* invades normally sterile sites such as the bloodstream.^2^ PCV7 provided protection against the seven most common serotypes causing IPD in the USA at the time, and countries have since adopted expanded PCVs that provide protection against serotypes estimated to cause approximately 70% of IPD globally.^3^ Today, 135 countries include a PCV in their national immunisation programme.^4^

In India, an estimated 105 100 (95% CI 92 100 to 120 000) of 356 300 (95% CI 311 600 to 407 400) under-five pneumonia deaths were associated with *S. pneumoniae* in 2010.^5^ In 2016, the National Technical Advisory Group on Immunization recommended introducing a PCV in the Universal Immunization Programme (UIP), which targets a cohort of 27 million newborns with six vaccines across the country and another two vaccines (against rotavirus and Japanese encephalitis) in a few states. The Indian government had planned to roll out PCV in three states in 2017, but progress remains slow in part due to the relatively high cost of PCV compared with other vaccines already provided under UIP.^6–8^ The Global Alliance for Vaccines and Immunization has pledged to support PCV provision until 2021,^9^ after which the cost of the vaccine will have to be borne by the Indian government. The affordability and cost-effectiveness of the vaccine is especially important in resource-constrained countries, such as India. Prior analyses in several HICs have found PCV introduction to be cost-saving or cost-effective according to WHO or local thresholds,^10–13^ but retrospective studies in other HICs, such as the Netherlands and Australia, found it unlikely that the PCV7 vaccination programme was cost-effective.^14 15^

In addition to PCV’s relatively high cost, the uncertainty regarding the vaccine’s potential cost-effectiveness in LMICs stems from uncertainty surrounding its effectiveness in these settings.^6 7 14^ The incidence of vaccine serotype (VT) IPD fell markedly in many HICs after the vaccine was introduced, but decreases in overall IPD varied significantly (eg, IPD decreased by 12% in Navarro, Spain while in the USA it decreased by 77%; see Weinberger *et al* 16). The increase in the frequency of *S. pneumoniae* serotypes not covered by the vaccine, also known as serotype replacement, contributed to this variation in overall IPD. In LMICs, evidence on the effect of serotype replacement on overall vaccine effectiveness in the population is lacking. Evidence on PCV from randomised control trials (RCTs) in LMICs demonstrated that PCV7 and PCV9 are highly efficacious at reducing pneumonia and invasive disease.^17–19^ However, RCTs are not designed to evaluate serotype replacement at the population level.

Recent observational studies of the introduction of a PCV in South Africa (PCV7 in 2009) and the Gambia (PCV13 in 2011) found that IPD in children under 2 dropped by 69% (95% CI 62 to 76) and 55% (95% CI 30 to 71) within 1 year of introduction, respectively. However, both studies found that disease caused by non-vaccine serotypes (NVTs) was increasing, and, in the case of the Gambia, overall IPD increased in the final year of the study.^20–22^ In southeast Asia, both Bangladesh and Nepal introduced PCVs in 2015, but data on effectiveness and serotype replacement have not been published.^4^

Economic analyses of introducing PCV need to consider the dynamics of *S. pneumoniae* transmission within the context of the setting being analysed. Local differences in distributions of dominant serotypes, host populations, health system structure and vaccination programmes all contribute to the variation in the vaccine’s impact across countries.^23^ To estimate PCV outcomes given these factors, models need to consider the colonised—asymptomatic carrier—population, which is the reservoir of transmission. In HICs that introduced PCV, serotype replacement among colonised individuals was higher and more consistent than serotype replacement in IPD. A logical explanation for higher replacement among the colonised individuals than in IPD cases is that NVTs cause less disease than VTs. If this is the case, the variation in reduced IPD across countries may be partially attributable to local differences in the distribution of colonising serotypes. Other theories have been proposed to explain the higher serotype replacement seen in colonisation than in IPD,^16^ and projecting the dynamics of *S. pneumoniae* transmission in LMICs with a paucity of data is difficult. Nonetheless, evaluations of PCV introduction need to consider these economic-epidemiological dynamics. They need to project serotype dynamics within the local context, or, at the very least, they should consider that the outcomes may not be the same in high-burden LMICs instead of current practice that either ignores the disease dynamics all together or assumes similar herd effects and serotype replacement as in low-burden–high-income settings (see^24–28^).

To project trends in under-five pneumococcal infections, including bacteraemic and non-bacteraemic pneumonia, meningitis and other IPD, and estimate the potential financial risk protection, cost and cost-effectiveness of introducing PCV in the UIP, we modelled *S. pneumoniae* dynamics in an agent-based model (ABM) of the Indian population and healthcare system.

## Methods

### Agent-based simulation model

We adapted our survey-data-driven ABM of an in silico population representative of the Indian population, IndiaSim.^29–32^ Our simulated population size was approximately 25 000 individuals and 4300 households. Individuals in the simulation interacted with each other (contacts) and with the healthcare system, getting vaccinated and seeking care. Individuals were either healthy and not colonised, healthy and colonised, or colonised and symptomatically infected. Those symptomatically infected with *S. pneumoniae* chose whether to seek care. Individuals could also seek care for exogenous infections. Simulations were run with 1-week time steps. Demographic and socioeconomic data and healthcare choices at the individual and household levels were drawn from the District Level Household Survey (DLHS-3) of India^33^ and from literature on care-seeking behaviour in India.^34 35^ Additional details on IndiaSim are in the online supplementary appendix and in previous publications.^30–32^ The model was programmed in C++11 standard and outcomes analysed in R, V.3.2.^36^

10.1136/bmjgh-2017-000636.supp1 Supplementary data

### Pneumococcal colonisation and transmission dynamics

Pneumococcal disease dynamics were included in IndiaSim based on work by Cobey and Lipsitch.^37^ We included 15 serotypes that are representative of the serotype distribution in India^38^; we did not model particular serotypes, but a representation of the *S. pneumoniae* population.

Transmission between individuals could occur when a carrier (or symptomatically infected) individual came into contact with other individuals. The probability of transmission of serotype z depended on the susceptibility of the individual, a function of both current and historical colonisations and infections:

(1) $$q(z,\vec{\theta },\vec{C})=\left[1-\omega (\vec{C})\right]\left[1-min\left(\left(1-p),min(1,\sigma \cdot \tau (z)\right)\right)\right]$$

where $\vec{\theta }$ and $\vec{C}$ are indicator vectors of past and current colonisation, indexed by z. Current colonisation was assumed to reduce susceptibility through competition, described by the term in the first bracket in (1), where $\omega (\vec{C})$ was set to:

(2) $$\omega \left(\vec{C}\right)=\left\{ \begin{array}{rl} 0, & \sum C_{i}=0\;(not\;colonized)\\ \mu_{max}\left[1-\frac{min\left(\vec{f}\right)-1}{Z-1}\right], & \sum C_{i}>0\;(colonized), \end{array}\right.$$

where Z is the number of serotypes in the model, $\mu_{max}$ is the maximum scaling down of susceptibility due to strain competition and $\vec{f}$ is a vector of serotype fitness ranks such that $min(\vec{f})$ is the rank of the most fit carried serotype. Serotype-specific immunity, described in the term in the second bracket in (1), also reduced susceptibility: p is vaccine efficacy for the targeted serotypes, σ is an anticapsular immunity parameter (equivalent for all serotypes) and

(3) $$\tau \left(z\right)=\left\{ \begin{array}{ll} 0, & \theta_{z}=0\;(not\;previously\;cleared)\\ 1, & \theta_{z}>0\;(previously\;cleared) \end{array}\right.$$

The duration of colonisation in successful transmissions was drawn from an exponential distribution in which the mean was

(4) $$v\left(z\right)=k+\left[\gamma \left(z\right)-k\right]e^{-\varepsilon \sum_{i}\theta_{i}}$$

Serotypes were assumed to differ in their fitness, modelled as a reduction in the length of colonisation (γ(z)).^39 40^ Duration exponentially decreased with the sum of past colonisations ($\sum_{i}\theta_{i}$), describing the serotype-independent immunity. k is the minimum duration of colonisation and ϵ is a fitted shape parameter. Parameterisation of colonisation and transmission dynamics are based on Cobey and Lipsitch,^37^ which fits the functions to data from vaccine-naïve populations.

If the person sought care (either for *S. pneumoniae* infection or for an exogenous infection) and was prescribed antibiotics, duration was updated accordingly. Additional details of the dynamics are in the online supplementary appendix, and the model parameters are presented in table 1 and in the following text.

**Table 1:** Parameters

Description	Symbol*	Base-case (sensitivity values/distribution)	Source
Disease model			
No of serotypes	*Z*	15	
Under-five colonisation prevalence fitted to		40%	Authors’ assumption based on^41–48 50 65^
Contact rate	*β*	Fitted to under-five colonisation prevalence	
Immigration force of infection	*W*	1e−06	As in^37^
Intrinsic duration of carriage for serotype *z*	*γ(z)*	25–220 days (linearly increasing across serotypes)	As in^37^ and based on^66 67^
Reduction in susceptibility to *Pneumococcus* from carrying the fittest serotype	*μ_max_*	0.25	As in^37^
Reduction in susceptibility to a serotype conferred by prior carriage of that serotype	*σ*	0.5 (0.5, 0.8)	≥0.5 based on results for *Z*=15 in^37^
Shape parameter for the reduction in duration of carriage dependent on past colonisation	*ε*	0.1 (0.1, 0.25 and 0.4)	Based on^37^
Case–carrier ratio (pneumococcal pneumonia, meningitis and other invasive pneumococcal disease)		Fitted to disease incidence given colonisation prevalence	Based on^5 52 65 68^
Case fatality rate		Fitted to death rate	Based on^5 51 52 65^
Treatment			
Seek treatment		Wealth quintile I: 48%; II: 51%; III: 60%; IV: 66%; V: 75%	Based on^34 35 69^
Probability seek care at public provider (if seek care)		Wealth quintile I: 55% (triangular min=44%, max=66%, mode=55%); II: 51% (triangular 40%, 61%, 51%); III: 43% (triangular 35%, 52%, 43%); IV: 39% (triangular 31%, 47%, 39%); V: 26% (triangular 21%, 32%, 26%)	
Receive appropriate treatment at health provider		95%	Authors’ assumption
Inpatient meningitis cost			Based on^54 69 70^
Public providers		US$191 (triangular min=US$134, max=US$248, mode=US$191)	
Private providers		US$275 (triangular min=US$193, max=US$358, mode=US$275)	
Inpatient pneumonia cost			Based on^54 69 71^
Public providers		US$93 (triangular min=US$65, max=US$121, mode=US$93)	
Private providers		US$214 (triangular min=US$150, max=US$278, mode=US$214)	
Inpatient other pneumococcal disease cost			Based on^54 69^
Public providers		US$76 (triangular min=US$53, max=US$99, mode=US$76)	
Private providers		US$194 (triangular min=US$136, max=US$252, mode=US$194)	
Outpatient cost			Based on^54 69^
Public providers		US$7.55 (triangular min=US$5.30, max=US$9.80, mode=US$7.55)	
Private providers		US$9.47 (triangular min=US$6.60, max=US$9.80, mode=US$12.30)	
Unattended pneumonia cost		US$1.05 (triangular min=US$0, max=US$1.40, mode=US$1.05)	72
Antibiotics clear colonisation or symptomatic infection		50%	Authors’ assumption based on^73–75^
Exogenous antibiotic prescription rate (per day)		0.001327	Based on IMS Health MIDAS database
Vaccine			
PCV13 % of cases		Most common serotypes representing approximately 70%	Based on^38 56^
Per-person vaccine efficacy	p	0.6	As in^37^, estimated using^76^
Per-child cost in scenario 1†		US$13.60 (triangular min=US$6.35, max=US$18.95, mode=US$13.60)	Based on WHO cMYP tool
Per-child cost in scenario 2†		US$13.50 (triangular min=US$6.25, max=US$18.85, mode=US$13.50)	Based on WHO cMYP tool

Values varied for sensitivity are in parentheses. Costs in 2014 US dollars.

*Symbols for Cobey and Lipsitch 2012 model.

†Three doses at US$3.30 per dose and training, syringe, wastage costs (5% vaccine wastage rate and 10% syringe wastage rate) and a 25% buffer stock. Ranges for the sensitivity assume US$1 to US$5 per dose.

cMYP, comprehensive multiyear plan; PCV, pneumococcal conjugate vaccine.

### Fitted pneumococcal colonisation prevalence

Studies from the past 15 years found *S. pneumoniae* colonisation prevalence in India ranging from 6.5% to 70.0% in children and infants.^41–50^ We fit the contact rate (*β*) so that the colonisation levels of children under-five were ~40%.

### Pneumococcal disease

Carriers of *S. pneumoniae* became symptomatically infected—developed bacteraemic or non-bacteraemic pneumococcal pneumonia, pneumococcal meningitis or other IPD—according to the invasiveness, or the case–carrier ratio. The case–carrier ratio represents infections per acquisition event, which we model as a function of the probability of progressing to symptomatic disease in a time step and the duration of carriage (the number of time steps). We assumed that VTs were carried for longer than NVTs,^40^ and therefore VT case–carrier ratio was greater. We fit the case–carrier and case–fatality rates to estimates of disease incidence and deaths in the literature.^51 52^ For more detail, see online supplementary appendix.

### Treatment and antimicrobial prescription

Individuals suffering from pneumococcal disease sought care (ie, went to hospital/clinic and received antibiotics) depending on their household wealth—wealthier individuals were more likely to seek care.^34 35^ Similar to Kouyos and others,^53^ we assumed that rates of colonisation were affected by individuals consuming antibiotics exogenously (ie, for other causes). Antibiotic consumption rates were drawn from IMS Health MIDAS (IMS Health, Danbury, Connecticut, USA) data on antibiotic consumption in India. The treatment costs for pneumococcal disease were based on Tasslimi et al^54^ and include care seeking, diagnostics, hospitalisation and medication.

### Vaccination scenarios

We evaluated three scenarios: (1) no vaccination; (2) introducing PCV13 at DPT3 (diphtheria, pertussis, tetanus vaccine) coverage levels (approximately 77%) and following the 2+1 schedule that India has adopted; and (3) increasing PCV13 coverage to 90%. We assumed that households that vaccinate with DPT in DLHS-3 continue to do so. We also increase coverage to 2011 estimates^55^; see previous work on rotavirus vaccination.^30^ For the extended vaccination scenario, additional households were recruited randomly to increase vaccination coverage rates to 90%.

The simulated vaccine did not protect against 13 simulated serotypes. Instead, we assumed the vaccine provided protection against the most common serotypes that contributed 70%–75% of disease incidence prior to vaccination^38 56^; this corresponded to 5 to 10 simulated VTs, depending on the simulation parameterisation. The vaccine was assumed to reduce susceptibility to asymptomatic carriage for VTs^37^ as described by equation (1). The vaccine likely further protects against carriers progressing to disease, but due to lack of evidence, we conservatively assumed that the vaccine only affects susceptibility to colonisation for covered serotypes and has no further effect on progression to disease (case–carrier ratio).

Data on immunisation costs were from India’s comprehensive multiyear plan for immunisation.^57^ It included costs for the vaccine and syringes—including wastage—and other related costs such as planning, training, transportation and cold chain equipment.

### Analysis and outcome measures

The primary outcome tracked was the change in under-five disease burden measured by estimated disease incidence and deaths averted. We report values for non-severe and severe pneumonia, pneumococcal meningitis and other IPD. We consider both bacteraemic and non-bacteraemic pneumonia, and the classification of severe pneumonia is based on the WHO definition used in Rudan *et al* 58 of lower chest wall indrawing, which represents an indication for hospitalisation. To measure serotype diversity, we calculated the Simpson index—the probability that two randomly selected serotypes (with replacement) will differ—and compared it with the limited data from India. We also estimated the years of life lost (YLLs) averted, the incremental cost-effectiveness (ICER) measured by the incremental cost per YLL averted from a health systems perspective (costs described above), out-of-pocket (OOP) expenditures averted and the money-metric value of insurance (VOI)—the dollar amount the population would be willing to pay to avert the risk of financial shock from OOP expenditure on treatment.^59^

We ran simulations with fitted values for the contact rate, case–carrier ratio and case–fatality rate for a 200-year burn-in period, before introducing vaccination and then estimating outcomes for the next 20 years. We report the rounded median present value for the 20-year intervention timeframe and annual outcomes. For averted burden estimates, we report differences between median values for each scenario; for example, to estimate the deaths averted by the intervention in scenario one, we subtract the median deaths in intervention scenario one from the median deaths in the no vaccination scenario. Costs and expenditures were converted to 2014 US dollars (see online supplementary appendix), and we used a discount rate of 3%, consistent with standard practice.

### Sensitivity analysis

In addition to the base-case analysis, to assess the sensitivity of our results, we varied the parameters for anticapsular immunity and serotype-independent immunity as described in Table 1 since the interplay between naturally acquired and vaccine acquired immunity likely impacts strain dynamics and serotype replacement. We ran simulations with each parameter set (and fitted contact rate, case–carrier ratio and case–fatality rate as described above), in total running 1800 simulations, 600 for each scenario. We constructed 95% CIs by drawing 5000 bootstrap samples (eg, of size 100 for base-case scenario 1 outcomes) from these simulations for each statistic we estimated. In addition, we explored the sensitivity of the ICERs to the immunity and economic parameters (Table 1); we set immunity parameters as described above and drew 5000 samples from the joint distribution of the economic parameters. We constructed cost-effectiveness acceptability curves by calculating the proportion of bootstrap samples that had the highest net benefit for each arm, where the net benefit =λ×\UpdeltaYLL−\Updeltacosts and λ is the willingness to pay per YLL.

## Results

### Serotype diversity

To compare the serotype diversity in our model with results in Cobey and Lipsitch^37^ and to data from India, we measured the Simpson Index for our model outcomes and compared it with the 0.93 index calculated from data collected by Manoharan *et al*,^56^ which identified 57 different serotypes and five non-typeable isolates. In our no-vaccination simulations, the median Simpson Index was 0.92 (95% CI 0.90 to 0.93).

### Disease burden

**Figure 1:**
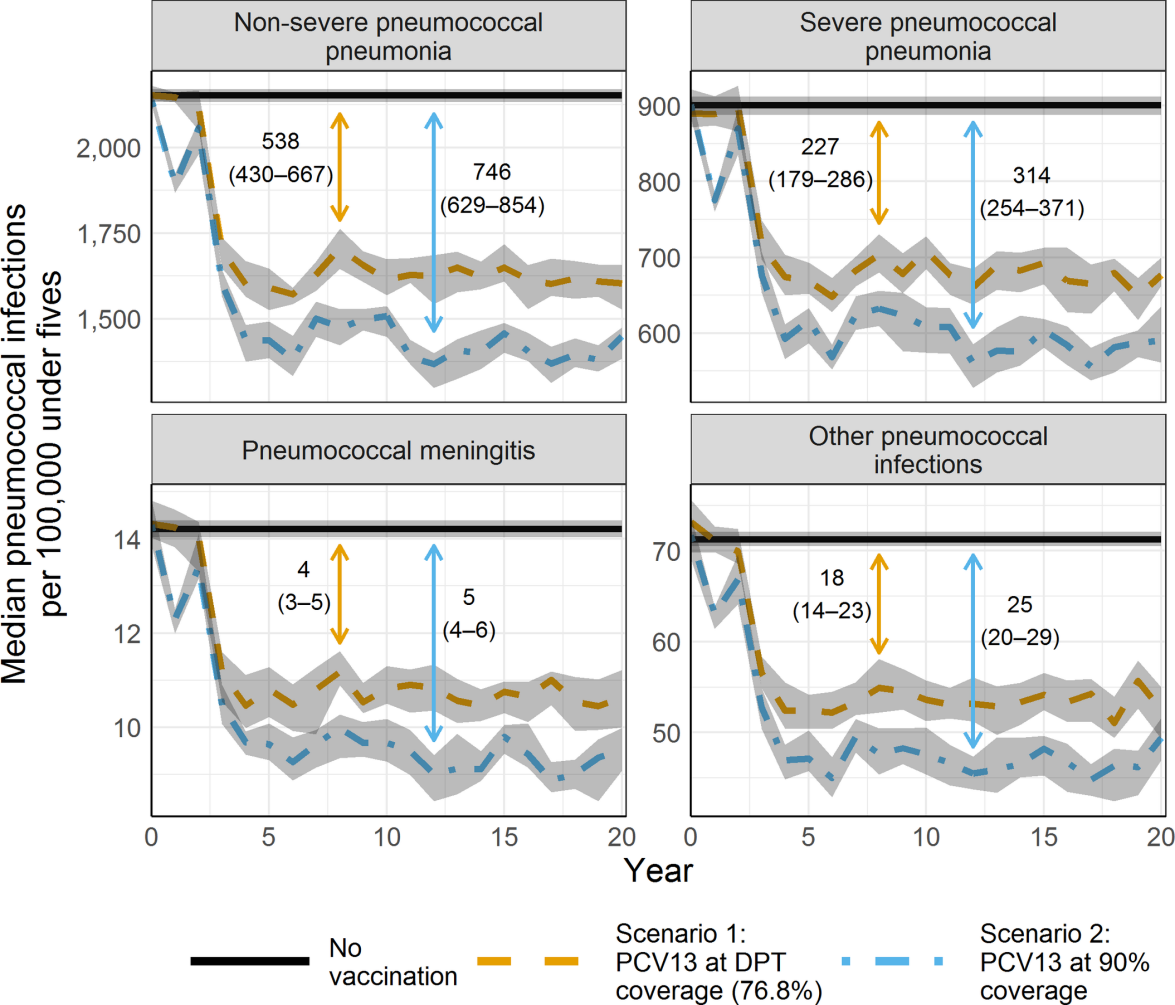
Pneumococcal disease cases (base case). Median pneumococcal disease incidence by year for 5000 bootstrap samples using base-case parameters. The line representing the no-vaccination scenario is the median across all years. The shaded areas represent the 95% CI for each year. The vertical lines with arrows and the corresponding values are the median cases averted after year 5, and the values in parentheses are the 95% CIs. DPT, diphtheria, pertussis and tetanus; PCV, pneumococcal conjugate vaccine.

We estimated that introducing PCV13 at current DPT coverage levels would avert a median 481 (95% CI 456 to 502) non-severe pneumonia cases, 198 (95% CI 185 to 211) severe pneumonia cases, 3 (95% CI 3 to 4) meningitis cases and 16 (95% CI 14 to 17) other invasive pneumococcal infections per 100 000 children under-five per year in the base case (figure 1). This represented a decline of 20.9% (95% CI 19.8% to 22.1%) in severe pneumococcal pneumonia cases per year. The number of cases only stabilises after 5 years, when it was 25.2% (95% CI 24.2% to 26.3%) and 34.2% (95% CI 31.9% to 36.7%) lower per year in the DPT and extended coverage scenarios than in the baseline scenario. Cases of non-severe pneumonia, meningitis and other invasive pneumococcal disease were similarly reduced.

**Figure 2:**
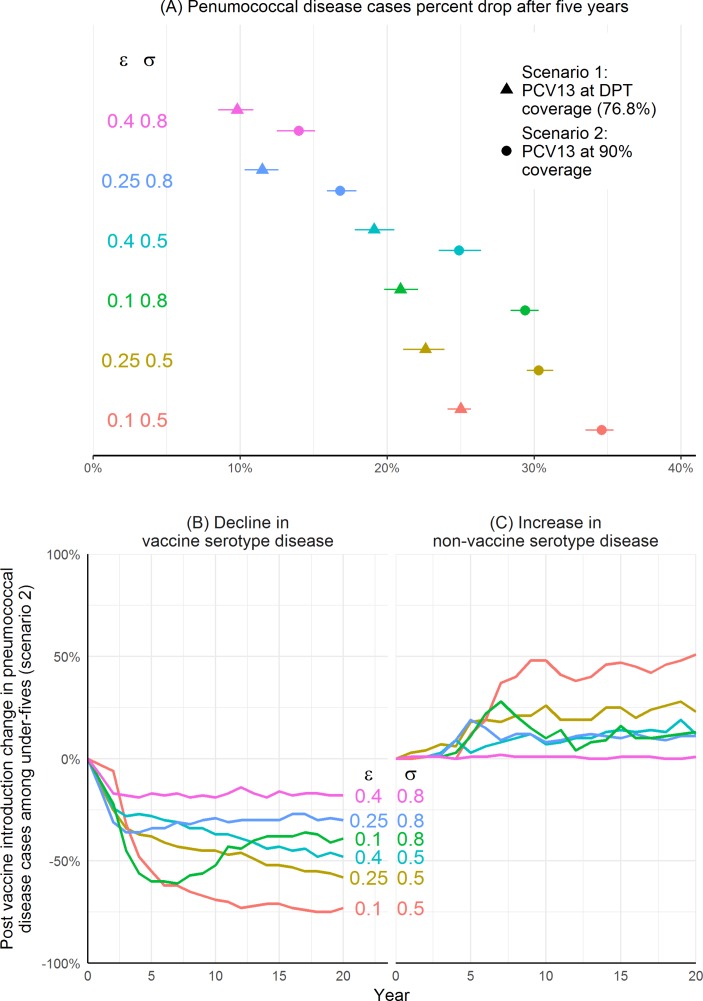
Sensitivity to immunity parameters. Sensitivity of pneumococcal disease cases, including non-severe and severe pneumococcal pneumonia, pneumococcal meningitis and other invasive pneumococcal infections, to immunity parameters over 5000 bootstrap samples. Panel (A) shows estimated cases averted per year for each parameter set. It is calculated by subtracting the median cases in scenario 1 and median cases in scenario 2 from the median cases in the no-vaccination scenario for each bootstrap sample. Dots and triangles are the predictions and line ranges are the 95% CIs. The other panels show serotype replacement over time; plotted values are the medians for each year. Panel (B) shows the per cent reduction in vaccine type cases of pneumococcal disease and (C) the per cent increase in non-vaccine-type pneumococcal cases after the introduction of PCV13% to 90% of the population (scenario 2). ϵ: Serotype-independent immunity shape parameter (see equation 4). σ: Anticapsular (serotype-specific) immunity parameter (see equation 1). DPT, diphtheria, pertussis and tetanus; PCV, pneumococcal conjugate vaccine.

Our results varied significantly depending on the sensitivity to immunity parameters, which affected the decline in under-five cases caused by VTs and serotype replacement by NVTs (figure 2A). In DPT vaccination coverage simulations where we set the serotype-specific immunity parameter, which impacts susceptibility, to the base-case value *σ*=0.5 (see equation 1) and increased the impact of serotype-independent immunity on colonisation duration from the base case by setting *ϵ*=0.25 or *ϵ*=0.4 (see equation 4), the number of cases dropped by 22.6% (95% CI 21.1% to 23.9%) and 19.1% (95% CI 17.8% to 20.5%), respectively. In simulations where we set the impact of serotype-specific immunity and serotype-independent immunity to the highest in our range (*σ*=0.8 and *ϵ*=0.4), the number of cases dropped by 9.8% (95% CI 8.5% to 10.9%).

VT symptomatic infections decreased and NVT symptomatic infections increased after the introduction of PCV13 for most parameter sets; there was no replacement by NVTs when immunity parameters were high (*σ*=0.8 and *ϵ*=0.4) (figure 2B and C). The highest increase in NVTs was in simulations with low immunity parameter values; by the end of expanded coverage simulations, NVT cases increased by 50.8% (95% CI 45.0% to 57.0%) and VT cases decreased by 73.1% (95% CI 71.8% to 74.2%) among under-fives when immunity parameters were low (*σ*=0.5 and *ϵ*=0.1). The decline in VT cases was lower when we increased the anticapsular immunity parameter, *σ*, than when we increased the serotype-independent immunity parameter, *ϵ*, and held other parameters at the base case. For example, when *σ*=0.5 and *ϵ*=0.4, VT cases decreased by 47.9% (95% CI 46.5% to 50.3%), and when *σ*=0.8 and *ϵ*=0.1 VT cases decreased by 39.0% (95% CI 36.9% to 42.0%) by the end of simulation. However, the increase in NVT cases was similar in these simulations: when *σ*=0.5 and *ϵ*=0.4, NVT cases increased by 12.1% (95% CI 9.2% to 17.8%), and when *σ*=0.8 and *ϵ*=0.1, NVT cases increased by 12.7% (95% CI 7.8% to 20.1%) by the end of simulation. Dynamics over time of VT decline differed when *σ*=0.8, which is higher than the vaccine’s serotype-dependent protection, p=0.6; before stabilising, VT disease increased slightly after the initial decline.

The estimated median number of deaths averted by PCV13 over 20 years was proportional to symptomatic infections (table 2). There were 558 (95% CI 457 to 656) deaths averted per 100 000 under-fives over 20 years in the DPT level vaccine coverage scenario in the base case, which, extrapolated to the full population, suggests 34 800 (95% CI 29 600 to 40 800) deaths averted in children under-five per year (the CIs in this case and for other extrapolations to the entire population do not account for uncertainty of the population size). We estimated that an additional 13 800 (95% CI 5600 to 19 000) deaths would be averted per year with expanded coverage. However, outcomes for different parameter sets varied significantly: when immunity parameters were the highest in our range (*σ*=0.8 and *ϵ*=0.4), the difference in median deaths averted per year was 11 000 (95% CI 5400 to 17 100) in the DPT level vaccine coverage scenario and 16 200 (95% CI 10 200 to 21 900) in the extended vaccine coverage scenario.

**Table 2:** Twenty-year outcomes and present value costs per 100 000 under-fives by wealth quintile (all parameter sets)

	I—poorest	II	III	IV	V—richest	Total
Scenario 1: PCV13 at DPT coverage (76.8%), incremental to the no-vaccination scenario						
Deaths averted	178 (127 to 226)	135 (89 to 184)	116 (66 to 151)	89 (32 to 122)	45 (19 to 87)	558 (457 to 656)
OOP expenditure averted*	US$143 (US$133 to US$154)	US$109 (US$102 to US$120)	US$83.7 (US$71.5 to US$93.4)	US$90.7 (US$80.4 to US$101)	US$111 (US$97.9 to US$122)	US$538 (US$514 to US$562)
Money-metric VOI*	US$78.0 (US$70.8 to US$84.4)	US$36.3 (US$33.7 to US$39.7)	US$20.6 (US$17.6 to US$23.4)	US$16.2 (US$14.0 to US$17.9)	US$8.90 (US$7.80 to US$10.0)	US$160 (US$151 to US$168)
Scenario 2: PCV13 at 90% coverage, incremental to scenario 1						
Deaths averted	55 (11 to 103)	78 (42 to 115)	16 (−17 to 58)	−5 (−37 to 42)	38 (−2 to 60)	186 (100 to 272)
OOP expenditure averted*	US$51.7 (US$41.2 to US$60.7)	US$37.2 (US$29.5 to US$44.7)	US$50.5 (US$42.1 to US$59.8)	US$32.9 (US$24.3 to US$43.4)	US$42.2 (US$33.7 to US$55.6)	US$215 (US$195 to US$237)
Money-metric VOI*	US$27.9 (US$22.1 to US$33.7)	US$12.5 (US$9.80 to US$15.3)	US$13.0 (US$10.7 to US$14.4)	US$5.90 (US$4.30 to US$7.80)	US$3.40 (US$2.60 to US$4.70)	US$62.6 (US$55.6 to US$69.6)

Incremental differences between medians for 20 simulated years in each scenario using base-case parameters: intervention scenario 1 incremental to the no-vaccination scenario and intervention scenario 2 incremental to scenario 1. The totals are for a population of 100 000 under-fives. The distribution of under-fives across wealth quintiles is not equal. 95% CIs in parentheses were constructed by 5000 bootstrap samples for each scenario overall all parameter sets.

*Present value discounted at 3% annually; US 2014 dollars; in thousands.

DPT, diphtheria, pertussis and tetanus; OOP, out-of-pocket; PCV, pneumococcal conjugate vaccine; VOI, value of insurance.

Deaths were inversely related to wealth. In the poorest portion of the population, 178 (95% CI 127 to 226) deaths were averted per 100 000 children under-five over the 20-year intervention assuming DPT vaccine coverage levels. An additional 55 (95% CI 11 to 103) deaths per 100 000 were averted when coverage was increased. The deaths averted in wealth quintiles IV and V, the wealthiest 40% of the population, were significantly lower than in the poorer population (89 (95% CI 32 to 122) in quintile IV and 45 (95% CI 19 to 87) in quintile V) at DPT coverage levels. Expanded coverage in these groups was not significantly different from no effect with an estimated −5 (95% CI −37 to 42) additional deaths averted in quintile IV and 38 (95% CI −2 to 60) in quintile V.

### Financial risk protection

We found that introducing PCV13 into the UIP protected households from the risk of expenditure on treatment and hospitalisation for pneumococcal diseases. The estimated base-case present value OOP expenditure averted per 100 000 was US$538 000 (95% CI US$514 000 to US$562 000) over 20 years at current vaccine coverage levels and an additional US$215 600 (95% CI US$195 000 to US$237 000) with expanded coverage (table 2). Extrapolating to the Indian population, after the fifth year of introducing PCV13, the median OOP expenditure averted would be approximately US$48.7 million annually under DPT vaccine coverage levels and an additional US$13.9 million with expanded coverage.

The median OOP expenditure averted was estimated to be highest for quintiles I (in the DPT coverage level scenario, the 20-year present value was US$143 000 (95% CI US$133 000 to US$154 000) per 100 000 under-fives in the base case), but it showed no clear trend across other wealth quintiles. The money-metric VOI decreased with wealth. The present value VOI was US$78 000 (95% CI US$70 800 to US$84 400) in wealth quintile I and US$8900 (95% CI US$7800 to US$10 000) in quintile V per 100 000 children under-five assuming DPT vaccine coverage levels. Increasing coverage provided additional protection, especially for wealth quintile I.

### Cost and cost-effectiveness

**Figure 3:**
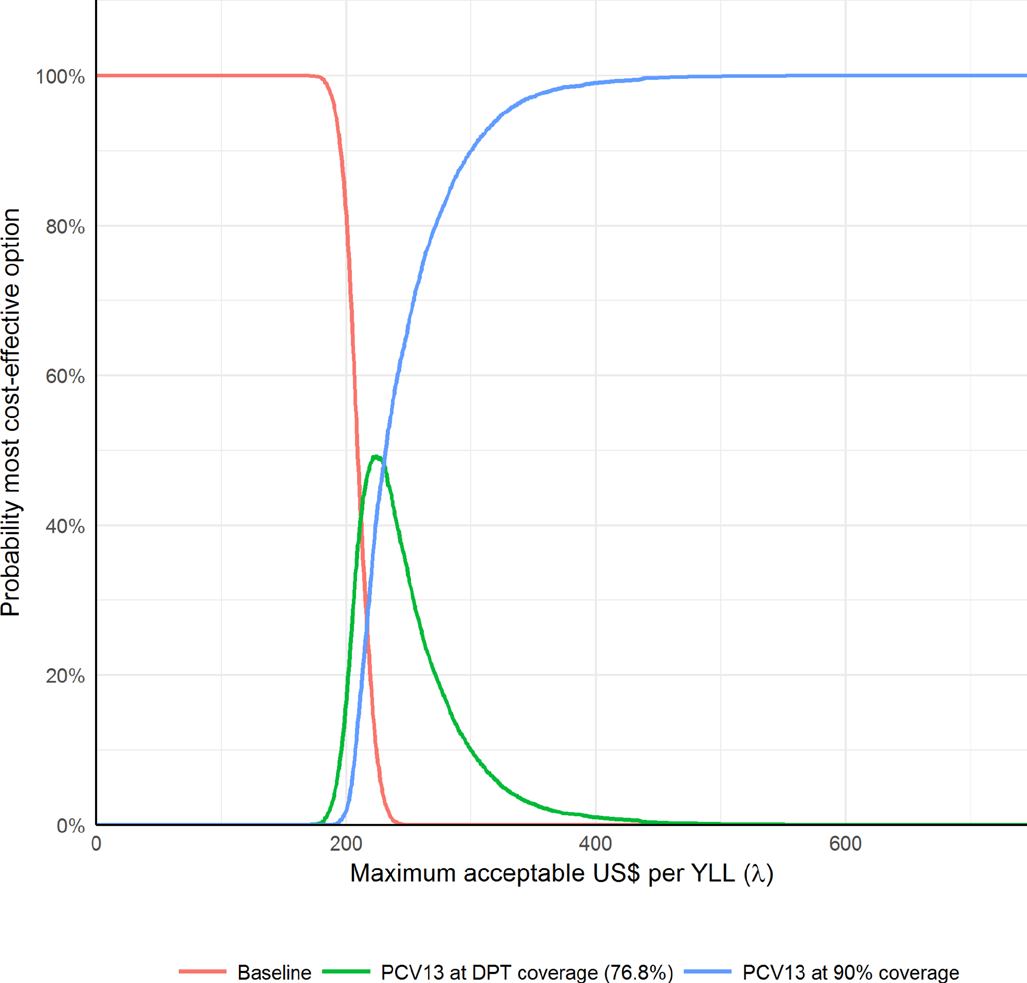
Cost-effectiveness acceptability curves. Cost-effectiveness from health system perspective. Includes all simulations. DPT, diphtheria, pertussis and tetanus; PCV, pneumococcal conjugate vaccine; YLL, years of life lost.

The present value cost of including PCV13 at DPT levels at US$3.30 per dose was approximately US$2.8 million per 100 000 and increasing coverage levels to 90% would increase this cost another US$1 million. Extrapolating to the population, the cost is approximately US$240 million each year under DPT coverage levels and US$328 million under expanded coverage. At US$1 per PCV13 dose, a similar cost to the rotavirus vaccine, the respective costs are approximately US$112 million and US$152 million per year. We estimated the median YLLs and calculated the cost per YLL averted. The incremental cost per YLL averted was US$144 under DPT vaccine coverage levels in the base case, and the incremental cost of expanding coverage was US$127. In the sensitivity analysis, the incremental cost per YLL averted was highest when immunity parameters where highest, reaching US$518 per YLL averted in the DPT vaccination coverage scenario. Figure 3 shows the cost-effectiveness acceptability curves for all simulations (including all parameter sets). We estimated that introducing the vaccine (scenario 1+scenario 2) is almost surely (in more than 95% of our simulations) more cost-effective than the baseline scenario f the willingness to pay per YLL averted, *λ*, is greater than US$228. If *λ* is greater than US$325, the extended coverage scenario is almost surely the most cost-effective option.

## Discussion

India’s recent decision to integrate the pneumococcal vaccine into its UIP is a response to the high pneumococcal disease burden in the country.^5^ The current cost of PCV is relatively high and its effectiveness uncertain given the paucity of information on asymptomatic carriage (the main reservoir of the bacteria), the distribution of IPD-causing serotypes in India^60^ and the potential changes to the serotype distribution after vaccine introduction. We examined these issues using an ABM. An ABM is helpful in this context as clinical trials are not feasible for predicting how a mass vaccination at the population level will affect serotype distribution. To that end, we simulated the effect of introducing the PCV13 vaccine into India accounting for differences in population wealth and access to health services.

We found that the introduction of PCV13 is likely to reduce the disease burden of *S. pneumoniae*. The greatest reduction in disease incidence and mortality is predicted to occur in the first few years after the introduction of the vaccine. This result is similar to other countries’ experiences and reflects the significant reduction in the most prevalent serotypes that are linked to the greatest incidence of disease.^61–64^ Though colonisation levels do not fall as precipitously, the new colonising serotypes are assumed to have a lower case–carrier ratio, which results in reductions in disease incidence and mortality. Our estimated per cent decline in disease incidence is modest compared with some studies in HICs,^61–64^ as well as in South Africa.^20^ This may be because of our conservative assumption that the vaccine does not explicitly impact disease incidence, but only affects it implicitly by reducing carriage of more fit serotypes. However, other factors contribute to the smaller effect on disease incidence. PCV7 serotypes contributed to a higher percentage of disease incidence in the pre-vaccine era in HICs (and PCV13 in South Africa^20^) than estimates of PCV13 serotypes contribute to disease in India.^38 56^ The impact of vaccination may be even smaller if the ABM population is not well-mixed—if we assume individuals are more likely to come into contact with others in their household or region (see online supplementary appendix). Because the vaccination coverage is heterogeneous in the DPT coverage scenario (according to existing DPT vaccination reported in DLHS-3), there may be unprotected pockets in the population. These pockets provide a reservoir for PCV13 strains and could propagate outbreaks of IPD with those strains.

The cost of implementing the vaccine is not insignificant; we estimated that it would cost at least US$240 million annually, more than double the estimated costs of implementing the rotavirus vaccine that India recently introduced.^30^ If PCV13 cost were to drop from US$3.30 per dose to US$1, a similar cost to the rotavirus vaccine and likely closer to the cost of a conjugate vaccine being developed in India, the annual cost would drop to approximately US$112 million. However, including the rotavirus vaccine in the UIP was estimated to reduce the disease and financial burden more than PCV13. The rotavirus vaccine was estimated to avert 44 500 deaths assuming DPT coverage,^30^ while the estimated number of median deaths averted by PCV13 is approximately 34 800 in our base case.

The estimate of US$144 per YLL in the DPT coverage scenario is a range that would be considered cost-effective. If willingness to pay per YLL is over US$325, introducing the vaccine with coverage extended to 90% was the most cost-effective option in over 95% of our bootstrap samples. The cost-effectiveness ratios in our analysis are in line with other projections in LMIC studies,^24 25 27^ but are higher than studies in Uganda (cost-saving at US$0.15 per dose)^26^ and in Kenya (mean US$47 per disability-adjusted life year at US$3.50 per dose).^28^ In addition to assuming different vaccine costs, these studies vary significantly from ours. For example, the study in Uganda does not consider serotype replacement, and the study in Kenya assumes replacement will be similar to the USA. In addition, we assumed the vaccine had no impact on the case–carrier ratio. If we altered that assumption, the vaccine’s effectiveness and cost-effectiveness would be greater.

Our study has a number of limitations. First and foremost, our estimates are uncertain, which is a reflection of the uncertainty in the parameters, particularly the efficacy of the vaccine to reduce the incidence of IPD as well as baseline rates of infection and mortality. Uncertainty is also partially a function of the size of the simulated population, which was ~25 000, with children under 5 representing 3000–4000 members of the population. We chose this population size to focus on a model of serotype dynamics that includes several serotypes. Our analysis does not fully capture the structural uncertainty of the disease model. We vary assumptions on the impact of immunity but maintain a similar model structure across simulations. Additionally, our demographics are based on sampling frameworks of the population. Though representative, they do not fully capture the heterogeneity that exists in a population as large as India. Our model does not currently consider sensitivity to the vaccine dose schedule, and we assume the 2+1 schedule rolled out in India.

Although the introduction of the PCV13 vaccine in India is likely to reduce the disease burden of *S. pneumoniae* and is cost-effective, the magnitude of the impact is uncertain. Data collection on pneumococcal carriage, disease and the prevalent serotypes in India and their virulence need to be strengthened. Filling these gaps while also increasing understanding of pneumococcal dynamics and reducing reliance on assumptions will improve our ability to project the serotypes likely to emerge and their impact on disease in India after introducing vaccination. Continuing surveillance after India introduces PCV will inform these dynamics as well, enhancing effective resource allocation and the success of future initiatives and course corrections. Though we caution that existing data gaps need to be filled, given our conservative assumptions, the disease and financial burdens averted and the relatively low expected cost per YLL saved make this an intervention worth pursuing.
