# Supplementary material for: Potential impact of introducing the pneumococcal conjugate vaccine into national immunisation programmes: an economic-epidemiological analysis using data from India
Source: BMJ Glob Health. 2018 May 9;3(3):e000636. doi: 10.1136/bmjgh-2017-000636 (PMC5950640; doi:10.1136/bmjgh-2017-000636)
Supplement: Supplementary data [file bmjgh-2017-000636supp001.pdf]

S1 Appendix

S1-1 IndiaSim agent-based simulation model structure

IndiaSim is an agent-based model (ABM) programmed in C++11 standard. The ABM was previously used to analyse the introduction of rotavirus vaccination in the UIP [1], expanding neonatal care by community health workers [2], universal public finance of epilepsy treatment [3], and implement water and sanitation interventions to reduce diarrheal diseases [4] among other analyses. The model is representative of the Indian population at the district level. It was constructed using the District Level Household Survey 2007–2008 (DLHS-3), which includes data on 34 Indian states (Nagaland is excluded), approximately 720,000 Indian households, and 3.4 million individuals. The survey reported information on individual characteristics (e.g., age, and sex) and household socioeconomic status. The survey also includes information health care facilities (e.g., their location and quality) and on households' care-seeking behaviour.

The ABM is structured in 67 patches, describing geographical units. Each patch is the urban or rural region in a state (Andaman and Nicobar's urban region is excluded because of small sample size). Each patch is populated by individuals, grouped into household units (for this analysis approximately 4,300 households were drawn from DLHS-3). Decisions in the model, including healthcare seeking and vaccination ones, are made at the household level. Households decide whether to vaccinate to protect from disease, and they decide whether to seek care when household members exhibit disease symptoms. For more details on the ABM see previous publications [1–3].

S1-2 Disease model

We modelled the dynamics of pneumococcal disease by building on a colonization model by Cobey and Lipsitch 2012 [5]. The model used similar assumptions, but was modified to include infection. At each time-step, a host carrying serotype z transmits to x individuals, where x is drawn from a Poisson distribution with a mean βI_z. β is the effective contact rate, a product of the host's contacts and likelihood of transmission, and I_z is the number of strains of serotype z the host carries. The x individuals the host transmits to are randomly picked from the entire population, where for each individual in the population the probability of being picked is weighted by their location relative to

the host and the parameter for fraction of contacts from within the same household (ρ_h), within the same patch (ρ_p), and neither $(1 - \rho_h - \rho_p)$.

Each of the x individuals acquires the strain based on their susceptibility. Similarly to Cobey and Lipsitch 2012 [5], host susceptibility to pneumococcal colonization by strain z is given by:

$$q(z, \vec{\theta}, \vec{C}) = [1 - \omega(\vec{C})]\left[1 - min\left((1-p), \min(1, \sigma \cdot \tau(z))\right)\right] \tag{1}$$

where $\vec{\theta}$ and $\vec{C}$ are vectors of past and current colonization indexed by z. The term in the second bracket of (1) represents naturally acquired and vaccine serotype-specific immunity. p is vaccine efficacy for the targeted serotypes. σ is an anticapsular immunity parameter (equivalent for all serotypes) and

$$\tau(z) = \begin{cases} 0, & \theta_z = 0 \text{ (not previously cleared)} \\ 1, & \theta_z > 0 \text{ (previously cleared).} \end{cases} \tag{2}$$

Host susceptibility is also a function of the serotypes currently carrying (the strain competition) represented by the term in the first bracket in (1). Competition is represented by

$$\omega(\vec{C}) = \begin{cases} 0, & \sum C_i = 0 \text{ (not colonized)} \\ \mu_{max}\left[1 - \frac{min(\vec{f}) - 1}{Z - 1}\right], & \sum C_i > 0 \text{ (colonized).} \end{cases} \tag{3}$$

where Z is the number of serotypes in the model, μ_{max} is the maximum scaling down of susceptibility due to strain competition, and $\vec{f}$ is a vector of serotype fitness ranks such that $min(\vec{f})$ is the rank of the most fit carried serotype.

The duration of a new colonization in a host is drawn from an exponential distribution with a mean

$$v(z) = k + [\gamma(z) - k]e^{-\epsilon \sum_i \theta_i} \tag{4}$$

where k is the minimum duration of colonization, $\gamma(z)$ is a serotype specific intrinsic colonization duration, and ϵ is a fitted shape parameter (see in [5] for fit). Duration exponentially decreases with the sum of past colonization, describing the non-specific immunity.

Carriers of strain z can become infected with the daily likelihood $\eta(z)$, which was equivalent across serotypes in our simulations, and infected individuals die according to the case-fatality rate.

S1-3 Simulation and fitting

Our estimates are based on a 20 year simulation time frame (in discrete time-steps of one week) for the three scenarios: the baseline scenario without PCV; scenario 1 with PCV13 coverage at households that also get DPT vaccination (approximately 77%); and scenario 2 with 90% PCV13 coverage. Each scenario was simulated using 6 different parameter sets: under-five colonization prevalence was set to 40%; the serotype specific immunity parameter, σ, was set to 0.5 or 0.8; and the non-specific immunity parameter, ϵ, was set to 0.1, 0.25, or 0.4; vaccination and treatment costs in public and private providers were also varied. These parameters are described further in the disease model section and the choice of their values is described in Table 1.

To initialize our populations' carriage and immunity distributions, we ran each parameter set for a 200 year burn-in period with no vaccination. We fit the simulations to three indicators: under-five colonization prevalence, the observed number of pneumococcal infections, and the number of deaths. Two sources estimated pneumococcal pneumonia, pneumococcal meningitis, and other invasive pneumococcal disease cases and mortality in India in the early 2000s [6,7], and two other studies have estimated these values for pneumococcal pneumonia in 2010 [8,9]. To achieve the fit, we did a parameter sweep, altering the contact rate (β), the daily rate of infection (for carriers), and the case fatality rate; we drew 100 samples for each of the 6 parameter sets using Latin Hypercube Sampling (LHS). We simulated each of the samples 10 times for a total of 6,000 simulations.

The best fits from the simulations used to initialize our populations were used as the starting point for simulations we used in the analysis. For this analysis, all three scenarios were simulated 100 times from each of the starting points using their respective values for σ, ϵ, and the fitted parameters. We then ran a total of 1,800 simulations (3 scenarios, 6 parameter sets, and 100 runs of each).

S1-4 Estimating outcomes

We estimated outcome measures and 95% CIs by drawing 5,000 bootstrap samples.

Incremental government expenditure and private, out-of-pocket expenditure averted

We considered cost of care-seeking, diagnostics, and treatment, including hospitalization and medication. These costs are out of pocket in all scenarios. Government expenditure is for introducing and including the pneumococcal conjugate vaccine in India's Universal Immunization Programme (UIP). These costs are from India's comprehensive multi-year plan (cMYP) for immunization [10] as described in the manuscript.

Using the outputs from IndiaSim we calculated the present day incremental values for both government costs and out-of-pocket (OOP) expenditure averted for each scenario. All costs were converted to 2014 US dollars using the Internal Revenue Service yearly average currency exchange rates [11] and GDP deflators [12].

Years of life lost (YLLs) and cost per YLL

We calculated the years of life lost YLLs averted using IndiaSim outputs on deaths due to pneumococcal infections. We also calculated the dollars-per-YLL averted. Costs included OOP expenditure and costs to the government for including the vaccine in the UIP. We discounted at 3% assuming uniform age-weights.

Money-metric value of insurance

We estimated the financial risk protection of averting out-of-pocket expenditure on care and treatment for individuals with pneumococcal infections. To estimate this we calculated the money-metric value of insurance, using a constant relative risk aversion utility function. For more information on this calculation please see Verguet et al. 2015 [13] and the appendix in Megiddo et al. 2016 [3].

References

1. Megiddo I, Colson AR, Nandi A, Chatterjee S, Prinja S, Khera A, et al. Analysis of the Universal Immunization Programme and Introduction of a Rotavirus Vaccine in India with IndiaSim. Vaccine. 2014;32: 151–161. doi:10.1016/j.vaccine.2014.04.080

2. Nandi A, Colson AR, Verma A, Megiddo I, Ashok A, Laxminarayan R. The Health and Economic Benefits of Scaling Up a Home-Based Neonatal Care Package in Rural India: A Modeling Analysis. Health Policy Plan. 2015;In Press.

3. Megiddo I, Colson A, Chisholm D, Dua T, Nandi A, Laxminarayan R. Health and economic benefits of public financing of epilepsy treatment in India: An agent-based simulation model. Epilepsia. 2016;57: 464–474. doi:10.1111/epi.13294

4. Nandi A, Megiddo I, Ashok A, Verma A, Laxminarayan R. Reduced burden of childhood diarrheal diseases through increased access to water and sanitation in India: A modeling analysis. Soc Sci Med. 2017;180: 181–192. doi:10.1016/j.socscimed.2016.08.049

5. Cobey S, Lipsitch M. Niche and neutral effects of acquired immunity permit coexistence of pneumococcal serotypes. Science. 2012;335: 1376–80. doi:10.1126/science.1215947

6. O'Brien KL, Wolfson LJ, Watt JP, Henkle E, Deloria-Knoll M, McCall N, et al. Burden of disease caused by Streptococcus pneumoniae in children younger than 5 years: global estimates. The Lancet. 2009;374: 893–902. doi:10.1016/S0140-6736(09)61204-6

7. Johnson HL, Bassani DG, Perin J, Levine OS, Cherian T, Brien KLO. Burden of childhood mortality caused by Streptococcus pneumoniae in India [Internet]. International Vaccine Access Center (IVAC) at the Johns Hopkins Bloomberg School of Public Health; 2012. Available: http://www.jhsph.edu/research/centers-and-institutes/ivac/resources/isppd8/H_Johnson-Burden_of_Child_Mortality_by_Strep_Pneumo_in_India.pdf

8. Fischer Walker CL, Rudan I, Liu L, Nair H, Theodoratou E, Bhutta ZA, et al. Global burden of childhood pneumonia and diarrhoea. The Lancet. 2013;381: 1405–1416. doi:10.1016/S0140-6736(13)60222-6

9. Farooqui H, Jit M, Heymann DL, Zodpey S. Burden of Severe Pneumonia, Pneumococcal Pneumonia and Pneumonia Deaths in Indian States: Modelling Based Estimates. PLoS One. 2015;10: e0129191. doi:10.1371/journal.pone.0129191

10. World Health Organization. WHO-UNICEF guidelines for developing a comprehensive multi-year plan (cMYP). In: WHO [Internet]. 2015 [cited 14 Nov 2015]. Available: http://www.who.int/immunization/programmes_systems/financing/tools/cmyp/en/

11. Internal Revenue Service. Yearly Average Currency Exchange Rates [Internet]. 2015 [cited 14 Oct 2015]. Available: https://www.irs.gov/individuals/international-taxpayers/yearly-average-currency-exchange-rates

12. Economy Watch. GDP Deflator Data for All Countries. In: EconomyWatch [Internet]. 2014 [cited 14 Oct 2015]. Available: http://www.economywatch.com/economic-statistics/economic-indicators/GDP_Deflator/

13. Verguet S, Laxminarayan R, Jamison DT. Universal Public Finance of Tuberculosis Treatment in India: An Extended Cost-Effectiveness Analysis. Health Econ. 2015;24: 318–332. doi:10.1002/hec.3019
